# Supplementary material for: Empirical methods for controlling false positives and estimating confidence in ChIP-Seq peaks
Source: BMC Bioinformatics. 2008 Dec 5;9:523. doi: 10.1186/1471-2105-9-523 (PMC2628906; doi:10.1186/1471-2105-9-523)
Supplement: Additional File 1 — A variety of html documents from the USeq web site detailing the available applications, their best usage, output file type descriptions, command line menus, etc. [file 1471-2105-9-523-S1.zip › USeqUserGuides/JavaDoc.zip!/JavaDoc/gata/aligner/class-use/LocalAlignment.html]

Uses of Class gata.aligner.LocalAlignment


|  |  |  |
| --- | --- | --- |
| **Overview** \| **Package** \| **Class** \| **Use** \| **Tree** \| **Deprecated** \| **Index** \| **Help** | |  |
| PREV   NEXT | **FRAMES**    **NO FRAMES**     **All Classes** |  |


---


## **Uses of Class gata.aligner.LocalAlignment**

| Packages that use LocalAlignment | |
| --- | --- |
| **gata.aligner** | For aligning and post processing DNA sequences using BLAST for subsequent import into GATAPlotter. |
| **gata.main** | Main app, utility, and params for GATAPlotter. |

Uses of LocalAlignment in gata.aligner

| Methods in gata.aligner that return LocalAlignment | |
| --- | --- |
| `LocalAlignment[]` | `Seqs.makeLocalAlignments(java.util.ArrayList data)` |

Constructors in gata.aligner with parameters of type LocalAlignment

`Alignment(java.lang.String s1,
int start1,
int stop1,
java.lang.String s2,
int start2,
int stop2,
int aScore,
int ori,
AlignParams ap,
LocalAlignment la)`

Uses of LocalAlignment in gata.main

| Methods in gata.main that return LocalAlignment | |
| --- | --- |
| `static LocalAlignment[]` | `GATAUtil.mergeLAArrays(LocalAlignment[] a, LocalAlignment[] b)` |

| Methods in gata.main with parameters of type LocalAlignment | |
| --- | --- |
| `static Alignment[]` | `GATAUtil.makeSubAligns(LocalAlignment[] locs)` |
| `static LocalAlignment[]` | `GATAUtil.mergeLAArrays(LocalAlignment[] a, LocalAlignment[] b)` |
| `static LocalAlignment[]` | `GATAUtil.mergeLAArrays(LocalAlignment[] a, LocalAlignment[] b)` |

---


|  |  |  |
| --- | --- | --- |
| **Overview** \| **Package** \| **Class** \| **Use** \| **Tree** \| **Deprecated** \| **Index** \| **Help** | |  |
| PREV   NEXT | **FRAMES**    **NO FRAMES**     **All Classes** |  |


---
